# Maintenance of effect of duloxetine in Chinese patients with pain due to osteoarthritis: 13-week open-label extension data

**DOI:** 10.1186/s12891-019-2527-y

**Published:** 2019-04-22

**Authors:** Guochun Wang, Liqi Bi, Xiangpei Li, Zhijun Li, Dongbao Zhao, Jinwei Chen, Dongyi He, Chia-Ning Wang, Tao Wu, Héctor Dueñas, Vladimir Skljarevski, Li Yue

**Affiliations:** 1 Rheumatology Department, China-Japan Friendship Hospital, Beijing, China; 2 Rheumatology Department, China-Japan Union Hospital of Jilin University, Changchun, Jilin, China; 3 Rheumatology Department, Anhui Provincial Hospital, Hefei, Anhui China; 4 Rheumatology Department, Affiliated Hospital of Bengbu Medical College, Bengbu, Anhui China; 5 Rheumatology Department, Shanghai Changhai Hospital, The Second Military Medical University, Shanghai, China; 6 Rheumatology Department, The Second Xiangya Hospital of Central South University, Changsha, Hunan China; 7 Rheumatology Department, Shanghai Guanghua Hospital, Shanghai, China; 8 Asian-Pacific Statistical Sciences, Lilly Suzhou Pharmaceutical Co. Ltd., Shanghai, China; 9 Medical Department, Lilly Suzhou Pharmaceutical Co. Ltd. Shanghai Branch, Shanghai, China; 10 Corporate Affairs Manager, Latin America Caribbean and Mexico, Eli Lilly de Mexico, Mexico City, Mexico; 11 Lilly Research Laboratories, Eli Lilly and Company, Indianapolis, Indiana, USA; 12 Medical Department, Lilly Suzhou Pharmaceutical Co. Ltd. Shanghai Branch, 19F, Centre T1, HKRI Taikoo, No. 288, Shimen No.1 Road, Shanghai, 200021 China

**Keywords:** Duloxetine, Osteoarthritis, Chronic pain, China

## Abstract

**Background:**

The objectives of this study were to assess the maintenance of effect of duloxetine 60 mg once-daily (QD) in Chinese patients with chronic pain due to osteoarthritis (OA) of the knee or hip and to provide additional long-term safety data.

**Methods:**

This was an open-label, extension phase of a randomized, double-blind, placebo-controlled clinical trial. Eligible patients were outpatients who met the American College of Rheumatology clinical and radiographic criteria for OA with a rating ≥4 on Brief Pain Inventory (BPI) 24-h average pain. After completing the 13-week placebo-controlled phase, patients originally assigned to placebo were titrated to duloxetine 60 mg QD (PLA_DLX), whereas patients originally assigned to duloxetine 60 mg QD remained on the same dose of duloxetine (DLX_DLX) for another 13 weeks. The maintenance effect of duloxetine 60 mg QD during the extension phase was evaluated by a 1-sided 97.5% confidence interval (CI) of the baseline-to-endpoint change in the extension phase for patients who took duloxetine and reported ≥30% reduction in BPI average pain at the end of placebo-controlled phase (placebo-controlled phase duloxetine responders). Other BPI severity and interference items, as well as safety and tolerability, were assessed.

**Results:**

Of 342 patients entering the extension phase, 162 (97.6%) DLX_DLX-treated patients and 157 (89.2%) PLA_DLX-treated patients completed this phase. Most patients (76.0%) were female. Mean age was 60.6 years. Mean BPI average pain was 5.5 at baseline of the placebo-controlled phase. Among 113 placebo-controlled phase duloxetine responders, mean change in BPI average pain during the extension phase was − 0.59 (from 2.47 to 1.88); the upper bound of the 1-sided 97.5% CI was − 0.31 and less than the pre-specified non-inferiority margin of a 1.5-point increase (*p* < 0.001). Significant within-group improvements in all BPI items were observed for both PLA_DLX and DLX_DLX groups during the extension phase (all *p* < 0.01). No deaths or suicide-related events occurred. Seven (4.0%) PLA_DLX-treated patients and no DLX_DLX-treated patients discontinued due to an adverse event.

**Conclusion:**

The analgesic effect of duloxetine 60 mg QD among treatment responders was maintained for the entire duration of the extension phase. Duloxetine 60 mg QD was well tolerated during the extension phase.

**Trial registration:**

ClinicalTrials.gov identification number NCT01931475. Registered 29 August 2013.

## Background

Osteoarthritis (OA) is a common degenerative joint disorder and its prevalence increases with age [1]. It is estimated that 9.6% of men and 18.0% of women aged ≥60 years have symptomatic OA worldwide [1]. Thirteen surveys involving 29,621 adults from six regions in China reported that the prevalence of OA ranged from 5.1 to 20.8%, with a mean of 9.1% [2]. In the 2010 World Health Organization Global Burden of Disease Study, OA was the eleventh leading cause of disability in the world and the sixth in East Asia [3]. Osteoarthritis can occur in any joint, with the knee, hip, and hand being most frequently affected [4]. Joint pain and loss of function are the predominant features of OA that lead to treatment [5]. The most commonly prescribed analgesics for OA pain include acetaminophen, nonsteroidal anti-inflammatory drugs (NSAIDs) (including cyclooxygenase-2 inhibitors), and opioids [6]. However, acetaminophen only provides minimal short-term benefit for people with osteoarthritis [7], and the long-term use of NSAIDs has been associated with an increased risk of serious gastrointestinal, cardiovascular, and renal harms [8, 9]. In addition, the use of opioids should be limited due to their serious risks, including sedation, respiratory depression, cognitive dysfunction, and serious constipation, with the risk of drug abuse and addiction posing an additional set of serious problems [10]. As a result, none of these drug classes are recommended for the long-term management of OA pain [11].

Although OA pain has traditionally been considered as peripheral/nociceptive pain that results from inflammation or mechanical damage in peripheral tissues, emerging evidence suggests that central sensitization is also an important mechanism underlying OA pain [12, 13]. Central sensitization involves the impaired activity of descending inhibitory pathway [14]. Serotonin (5-HT) and norepinephrine (NE) are key neurotransmitters in the descending inhibitory pathway and thus involved in pain modulation [15]. Duloxetine, as a potent and selective inhibitor of 5-HT and NE reuptake in the central nervous system [16], has been shown to be effective in four chronic pain conditions, including OA pain [17, 18], chronic low back pain (CLBP) [19–21], fibromyalgia [22–25], and diabetic peripheral neuropathic pain (DPNP) [26–28].

A double-blind, placebo-controlled, 13-week study of duloxetine treatment in Chinese patients with OA pain was recently completed. Results from this 13-week placebo-controlled study showed a significant improvement in both the primary outcome measure of pain severity and in most of the secondary outcome measures [29]. Patients completing the 13-week placebo-controlled phase were given the option of entering a 13-week, open-label extension phase [30]. The current article reports results from this extension phase study. The main objectives of the extension phase were to evaluate the maintenance of effect of duloxetine in patients with OA pain and to provide additional long-term safety data in this patient population. Pain severity was measured by the 24-h average pain ratings of the Brief Pain Inventory (BPI) (referred to as BPI average pain hereafter).

## Methods

### Study design

This was the 13-week, open-label, extension phase of a randomized, double-blind, multicenter, placebo-controlled trial (ClinicalTrials.gov NCT01931475) [29]. Patients taking duloxetine 60 mg once daily (QD) during the placebo-controlled phase remained on that dose during the extension phase (DLX_DLX group). Patients receiving placebo during the placebo-controlled phase took duloxetine 30 mg QD for 1 week followed by duloxetine 60 mg QD for 12 weeks during the extension phase (PLA_DLX group). Patients from both groups (DLX_DLX and PLA_DLX) who could not tolerate duloxetine 60 mg QD during the extension phase were discontinued from the study and entered the taper phase. In the taper phase, patients received duloxetine 30 mg QD for 1 week.

This extension phase study was conducted in accordance with consensus ethics principles derived from international ethics guidelines, International Conference on Harmonisation Guideline for Good Clinical Practice E6, and all applicable laws and regulations.

#### Patients

Eligibility criteria have been described previously [29]. The main inclusion criteria were male and female outpatients aged at least 40 years who met the American College of Rheumatology clinical and radiographic criteria for the diagnosis of OA of the knee or hip, had pain for ≥14 days of each month for 3 months before study entry, and had a rating of ≥4 on the BPI average pain item (on a 0 [no pain] to 10 [pain as severe as one can imagine] scale) during screening.

Patients were excluded from the trial if they were taking any excluded medications (analgesic agents including but not limited to NSAIDs, acetaminophen, and opioids).

Both the participants and investigators remained blinded to original treatment allocation when the patients entered into the extension phase. Concomitant use of analgesics and other medications was not restricted in the extension phase (except for the few which may pose drug-drug interaction risks) and depended on investigators’ judgment. However, no rescue medication (short-acting analgesics, such as acetaminophen, NSAIDs, and codeine, used for rescue from an OA knee or hip pain flare) was allowed during the 24 h prior to any study visit during the extension phase.

#### Efficacy and safety measures

The BPI–Severity and BPI–Interference scales were completed by the patient at each study visit to measure the severity of pain and the interference of pain on function during the previous 24 h before the scheduled visit. The average pain item of BPI-Severity was assessed as the primary efficacy measure. Other BPI–Severity items include worst pain, least pain, and pain right now. The BPI–Interference items include general activity, mood, walking ability, normal work, relations with other people, sleep, and enjoyment of life. Ratings of the BPI items range from 0 (no pain/does not interfere) to 10 (pain as severe as one can imagine/completely interferes) [31].

Safety measures assessed during the extension phase included treatment-emergent adverse events (TEAEs), serious adverse events (SAEs), the Columbia-Suicide Severity Rating Scale, assessment of falls using solicited questioning, standard laboratory assessments, vital signs, and discontinuation rates due to adverse events (AEs).

#### Statistical analyses

All analyses were conducted on an intent-to-treat basis. Statistical comparison between treatment groups was not performed for the extension phase. Within-group changes from baseline to endpoint were evaluated by two-sided t-test for efficacy measures and the Wilcoxon signed-rank test for safety measures. Unless otherwise stated, baseline and endpoint were defined as the last non-missing observation during the placebo-controlled phase and the last non-missing observation during the extension phase (last observation carried forward), respectively [32]. Changes were considered statistically significant at the 0.05 level, unless otherwise stated. Statistical Analysis System software (version 9.2) was used to perform all statistical analyses.

Patients who took duloxetine and reported a ≥30% reduction in BPI average pain during the placebo-controlled-phase were defined as placebo-controlled phase duloxetine responders. The maintenance of effect of duloxetine 60 mg QD during the extension phase was evaluated in placebo-controlled-phase duloxetine responders by a 1-sided 97.5% confidence interval (CI) of the baseline-to-endpoint change in the extension phase. At the significance level of 0.025 (one-tailed), the null hypothesis of non-maintenance was rejected, if the upper limit of the 1-sided 97.5% CI was a ≤1.5 point (non-inferiority margin) increase in BPI average pain. A non-inferiority margin of 1.5 points was selected based on previous long-term studies of duloxetine in DPNP [33] and CLBP [32] as well as a study of opioids in cancer pain [34].

In addition, a mixed-model-repeated-measures (MMRM) analysis was performed to analyze the change in BPI average pain for all patients who entered the extension phase using data collected during the entire study (both the placebo-controlled and the extension phases). Type III sum-of-squares for the least-squares means was used and the last non-missing observation during screening was considered as baseline.

The percentages of patients who achieved a ≥30% or ≥50% reduction in BPI average pain from baseline to endpoint were summarized. Here, the baseline and endpoint were defined as the last non-missing observation during screening and the last non-missing observation during the extension phase, respectively.

## Results

### Patient disposition

**Fig. 1:**
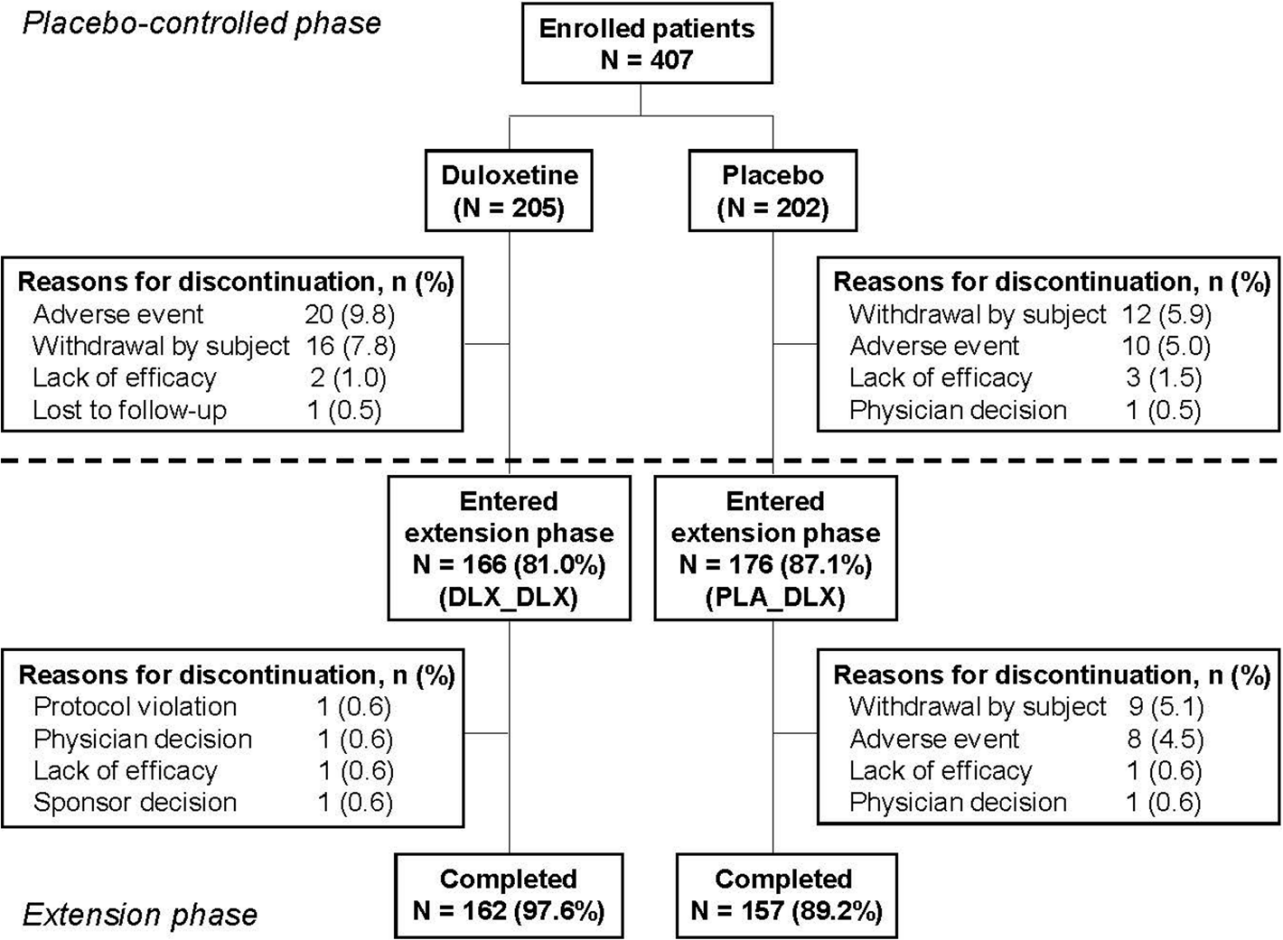
Patient disposition. Abbreviations: DLX_DLX = patients who received duloxetine during both the placebo-controlled and the extension phases; PLA_DLX = patients who received placebo during the placebo-controlled phase and duloxetine during the extension phase

From a total of 407 randomized patients, 39 patients from the DLX group and 26 patients from the PLA group discontinued the study during double-blind phase, and a total of 342 (166 in the DLX group and 176 in the PLA group) entered into the open-label extension phase [29]. Among them, 162 (97.6%) patients in the DLX_DLX group and 157 (89.2%) patients in the PLA_DLX group completed the extension phase (Fig. 1). Reasons for discontinuation in the DLX_DLX group during the extension phase included protocol violation, physician decision, lack of efficacy, and sponsor decision (1 [0.6%] patient each). The primary reason for discontinuation in the PLA_DLX group during the extension phase was withdrawal by subject (9 [5.1%] patients) followed by an AE (8 [4.5%] patients).

### Baseline demographic and clinical characteristics

Demographic and clinical characteristics were comparable between the groups (PLA_DLX and DLX_DLX) at baseline of the placebo-controlled phase (Table 1). All patients in the extension phase were Asian, and the majority (76.0%) of them were female. The mean age of the patients was 60.6 years. The majority of patients (341 [99.7%] patients) had OA of the knee, whereas only 1 (0.3%) patient had OA of the hip. Mean duration of pain due to OA was 7.69 years. Mean BPI average pain was 5.6 for the DLX_DLX group and 5.5 for the PLA_DLX group at baseline of the placebo-controlled phase, and 3.3 for the DLX_DLX group and 3.7 for the PLA_DLX group at baseline of the extension phase.

**Table 1:** Demographic and Clinical Characteristics at Baseline of the Placebo-Controlled Phase

Variable	DLX_DLX	PLA_DLX
	*N* = 166	*N* = 176
Age, years, mean (SD)	61.2 (8.0)	60.0 (8.3)
Gender, n (%)		
Female	130 (78.3)	130 (73.9)
BMI, kg/m^2^, mean (SD)	25.5 (3.2)	25.3 (3.6)
Location of OA, n (%)		
Hip	0	1 (0.6)
Knee	166 (100.0)	175 (99.4)
Duration of OA pain, years, mean (SD)	7.7 (6.6)	7.6 (6.9)
BPI 24-h average pain, mean (SD)	5.6 (1.3)	5.5 (1.2)
BPI–Interference average rating, mean (SD)	3.6 (2.0)	3.6 (1.9)

*Abbreviations*: *BMI* body mass index, *BPI* brief pain inventory, *DLX* duloxetine, *N* number of patients in group, *n* number of patients, *OA* osteoarthritis, *PLA* placebo, *SD* standard deviation

### Concomitant analgesics

Two (1.2%) DLX_DLX-treated patients and 2 (1.1%) PLA_DLX-treated patients were taking at least 1 short-acting analgesic during the extension phase. In the DLX_DLX group, 1 patient took acemetacin 90 mg/day for 8 days and 1 patient took diclofenac sodium 150 mg/day for 2 days. In the PLA_DLX group, 1 patient took loxoprofen 180 mg/day for 2 days and 1 patient took 3 units/day of paramol-118 for 3 days.

### Efficacy

A total of 113 patients in the DLX_DLX group met the 30% response criterion after the 13-week placebo-controlled phase. Among these patients, the mean BPI average pain changed from 2.47 to 1.88 during the extension phase (mean change: − 0.59; 1-sided 97.5% CI: -∞, − 0.31). The upper limit of the 1-sided 97.5% CI was significantly (*p* < 0.001) less compared to the non-inferiority margin, which was prespecified as 1.5-points increase on BPI average pain. This indicates that in those patients who responded to duloxetine during the placebo-controlled phase, the treatment effect of duloxetine 60 mg QD on pain reduction was maintained over the 13-week extension phase. In addition, since the upper bound of the 1-sided 97.5% CI was < 0, the pain severity was statistically significantly reduced during the extension phase versus the end of the placebo-controlled phase.

**Fig. 2:**
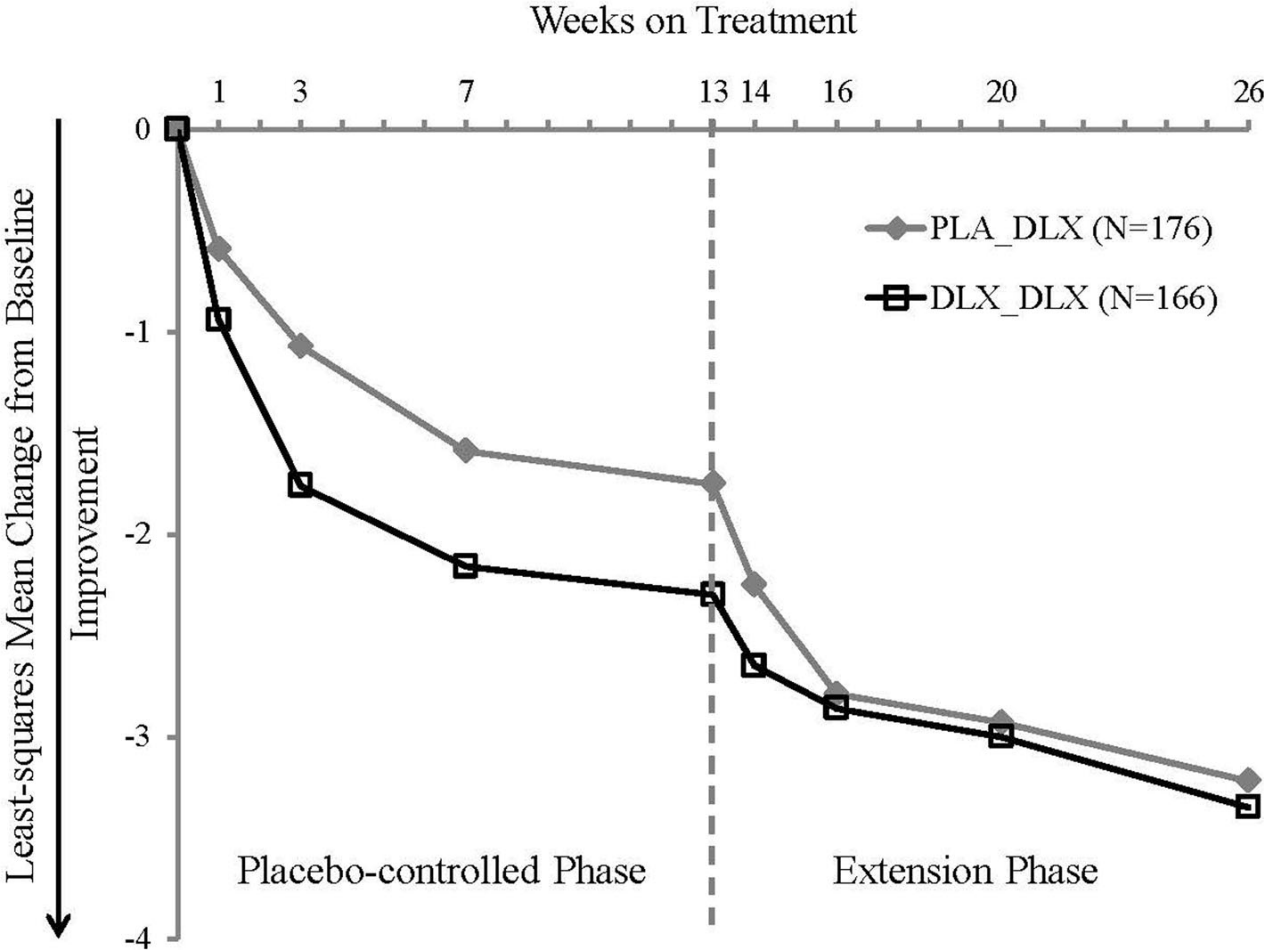
Least-squares mean change in BPI average pain rating for patients who entered the extension phase. Mixed-model repeated measures analysis. Abbreviations: BPI = Brief Pain Inventory; DLX = duloxetine; PLA = placebo

Figure 2 shows the change in BPI average pain rating for all patients who entered the extension phase, from baseline of the placebo-controlled phase through the end of the extension phase. Both PLA_DLX and DLX_DLX patients experienced continuous pain reduction during the entire 26-week study duration (placebo-controlled and extension phases).

**Fig. 3:**
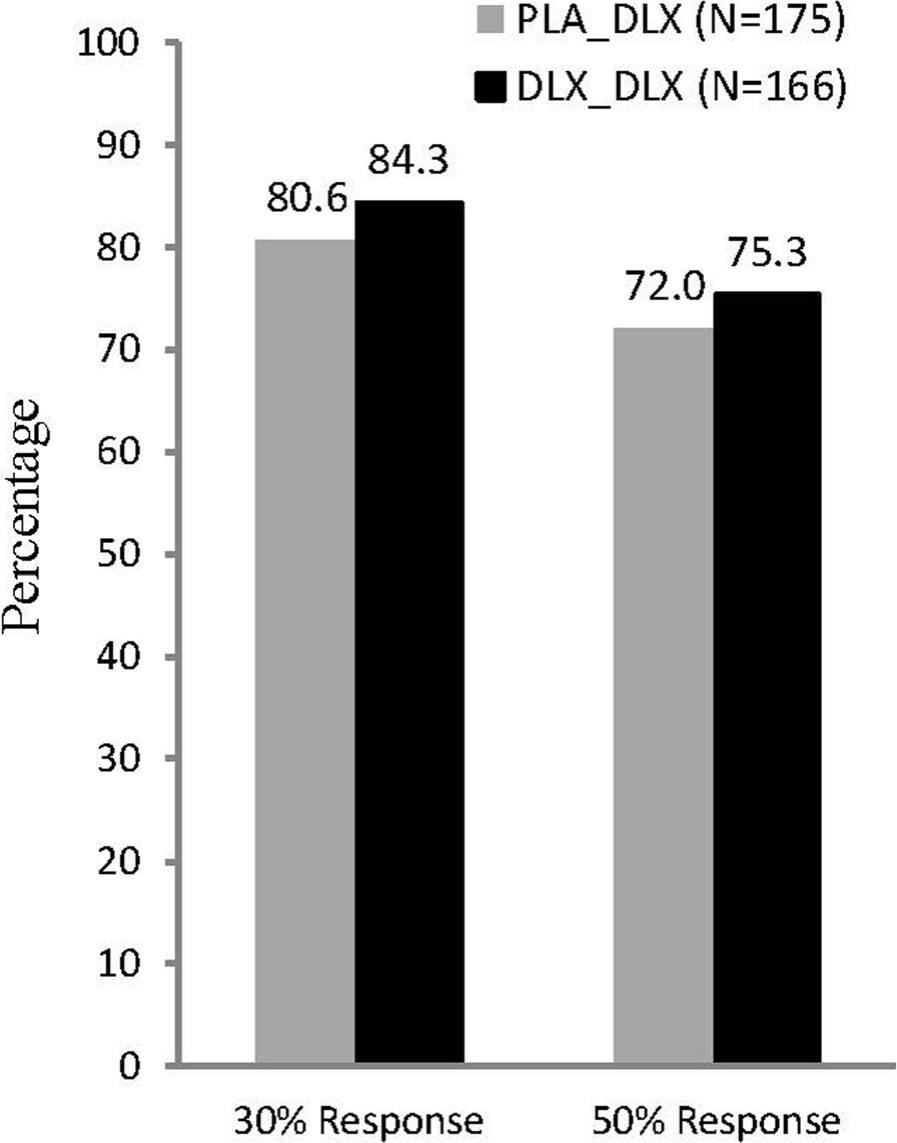
Response rates at the end of the extension phase. The response rates were based on the change from baseline of the placebo-controlled phase to endpoint (last observation carried forward) in Brief Pain Inventory average pain rating. Abbreviations: DLX = duloxetine; PLA = placebo

The majority of patients from both the PLA_DLX and DLX_DLX groups met the 30% response and 50% response criteria at the end of the extension phase (Fig. 3). Of the 113 placebo-controlled phase duloxetine responders, 105 (92.9%) met the 30% response criterion at the end of the extension phase, and 98 (86.7%) met the 50% response criterion.

Both the PLA_DLX and DLX_DLX groups showed significant within-group improvements during the extension phase on all other 3 BPI–Severity items (worst pain, least pain, and right now pain), BPI–Interference average rating, and all 7 individual BPI–Interference items (Table 2).

**Table 2:** Mean Change in BPI–Severity and BPI–Interference Items during the Extension Phase

Variable	DLX_DLX		PLA_DLX	
	N = 166		*N* = 175	
	Baseline	Change	Baseline	Change
Average pain	3.3 (1.8)	−1.0 (1.7)^***^	3.7 (1.8)	−1.4 (1.6)^***^
Worst pain	4.2 (2.1)	−1.1 (2.0)^***^	4.8 (1.9)	−1.6 (1.7)^***^
Least pain	2.2 (1.9)	−0.9 (1.6)^***^	2.5 (1.9)	−1.0 (1.7)^***^
Right now pain	2.8 (2.1)	−1.1 (1.7)^***^	3.3 (2.1)	−1.4 (1.7)^***^
Average Interference	1.9 (1.8)	−0.6 (1.1)^***^	2.2 (1.7)	−0.8 (1.2)^***^
General activity	3.0 (2.3)	−0.9 (1.9)^***^	3.6 (2.2)	−1.3 (1.9)^***^
Mood	1.2 (2.0)	−0.4 (1.2)^***^	1.6 (2.1)	−0.6 (1.7)^***^
Walking ability	3.0 (2.2)	−0.9 (1.6)^***^	3.5 (2.2)	−1.3 (1.7)^***^
Normal work	2.6 (2.3)	−0.9 (1.8)^***^	3.1 (2.2)	−1.1 (1.9)^***^
Relations with other people	0.8 (1.8)	−0.2 (1.1)^**^	0.9 (1.7)	−0.3 (1.2)^**^
Sleep	1.3 (2.2)	−0.5 (1.7)^***^	1.5 (2.1)	−0.5 (1.6)^***^
Enjoyment of life	1.2 (2.1)	−0.5 (1.5)^***^	1.2 (2.0)	−0.5 (1.6)^***^

^**^p < 0.01; ^***^p < 0.001 (within-group, 2-sided t-test). Data are shown as mean (standard deviation). Baseline is the last non-missing observation during the placebo-controlled phase. Change is the change from the last non-missing observation during the placebo-controlled phase to the last non-missing observation during the extension phase

*Abbreviations*: *BPI* Brief Pain Inventory, *DLX* duloxetine, *PLA* placebo

### Safety

Table 3 presents TEAEs experienced by at least 2% of patients in either treatment group during the extension phase. Overall, a greater percentage of PLA_DLX patients (46.3%) experienced at least 1 TEAE compared to DLX_DLX patients (25.3%). For PLA_DLX patients, the most frequently observed TEAEs were dry mouth, somnolence, and alanine aminotransferase (ALT) increased. For DLX_DLX patients, the most frequently observed TEAEs were nausea and somnolence.

**Table 3:** Treatment-Emergent Adverse Events^a^ Observed during the Extension Phase

Adverse Event, n (%)	DLX_DLX	PLA_DLX
	N = 166	N = 175
Patients with ≥1 TEAE	42 (25.3)	81 (46.3)
Dry mouth	3 (1.8)	15 (8.6)
Somnolence	4 (2.4)	10 (5.7)
Alanine aminotransferase increased	3 (1.8)	10 (5.7)
Nausea	5 (3.0)	8 (4.6)
Aspartate aminotransferase increased	1 (0.6)	8 (4.6)
Dizziness	3 (1.8)	7 (4.0)
Constipation	1 (0.6)	7 (4.0)
Weight decreased	0	4 (2.3)

*Abbreviations*: *DLX* duloxetine, *PLA* placebo, *TEAE* treatment-emergent adverse event

^a^TEAEs occurring at a rate ≥2% in either treatment group

No deaths or suicide-related events were reported during the extension phase. DLX_DLX patients did not experience any SAEs or discontinue the study due to an AE. Two (1.1%) PLA_DLX patients experienced four SAEs, including 1 incidence each of fall, lacunar infarction, skull fracture, and vertebrobasilar insufficiency. Seven (4.0%) PLA_DLX patients discontinued the study due to an AE. No AE led to study discontinuation in more than 1 patient, except for nausea in 2 patients. Taken together, these data suggest that the incidences of SAEs, AEs leading to discontinuation, and TEAEs including those commonly observed with duloxetine treatment, were lower in patients with long-term (6 months) exposure to duloxetine compared to those with short-term (3 months) exposure during the extension phase, although statistical comparison between treatment groups was not performed.

Seven (4.0%) PLA_DLX patients and 3 (1.8%) DLX_DLX patients reported at least 1 fall at the end of the extension phase. Five of the 10 patients reported an AE of fall or ligament sprain, among which one led to hospitalization and thus considered as an SAE. None of the AEs or SAE was related to the study drug or protocol procedures as per the investigators’ judgment.

Vital signs were stable relative to the end of the placebo-controlled phase. No patients in the PLA_DLX group had sustained (3 consecutive visits) elevations in either diastolic or systolic blood pressure, whereas 1 (0.6%) patient in the DLX_DLX group had sustained elevations in systolic blood pressure. Twenty-five (14.3%) PLA_DLX patients and 17 (10.2%) DLX_DLX patients experienced orthostatic hypotension.

No DLX_DLX patients had ALT ≥3 times upper limit of normal (ULN) during the extension phase. Three (1.9%) PLA_DLX patients had treatment-emergent ALT ≥3 times ULN, and 1 of them had ALT ≥10 times ULN. All 3 patients completed the study. Two of the 3 patients had their ALT levels decreased to < 2 times ULN and 1 patient had the ALT level return to normal at the end of the extension phase. No clinically relevant changes were observed for other chemistry analytes.

## Discussion

Duloxetine-treated patients with OA pain who were responders during the placebo-controlled phase benefited from continuing treatment with duloxetine 60 mg QD in the 13-week extension phase of this study. The upper limit of the group mean change in BPI average pain was lower than the priori-specified non-inferiority margin, demonstrating that the pain reduction from duloxetine treatment obtained at the end of the initial 13 weeks was maintained during the subsequent 13-week extension phase. From the individual patient level, of the 113 placebo-controlled-phase responders, 105 maintained ≥30% pain reduction at the end of the extension phase. Moreover, the upper limit of the group mean change in BPI average pain was less than zero, suggesting a statistically significant pain reduction from the end of the placebo-controlled phase to the end of the extension phase, thus further highlighting the importance of continuing duloxetine treatment over time.

Two previous duloxetine studies observed improvements or minimal worsening in pain measures in patients with fibromyalgia in the 6-mounth extension phases [35]. Pain relief was also maintained in patients with DPNP for 26 weeks and in patients with CLBP for 41 weeks [32, 33]. These studies and the present study together indicate that the maintenance of effect of duloxetine is consistent across chronic pain conditions.

Only two patients in each treatment group were taking at least 1 short-acting analgesic during the extension phase, which is much fewer than those observed in previous studies in DPNP and CLBP [32, 33]. One possible reason is that the duration of the extension phase in this study (13 weeks) is shorter than that of studies in DPNP (26 weeks) and CLBP (41 weeks). Alternatively, it may be because 11.2% of patients were taking herbal and traditional medicine and some of these medicines may have analgesic effects.

The safety and tolerability profile of duloxetine during the extension phase was comparable with that observed in the placebo-controlled phase of this study and in the previous studies of duloxetine for other chronic pain conditions [32, 33, 35]. None of DLX_DLX patients experienced any SAEs or suicide-related events or discontinued the study due to an AE. Overall, 25.3% of DLX_DLX patients experienced at least 1 TEAE, and the most frequently observed TEAEs among these patients were nausea and somnolence, both of which are established common AEs of duloxetine [36]. The results of cardiovascular assessments and chemistry analytes were also similar to previous studies of duloxetine for other indications [37, 38]. Taken together, these findings indicate that there is no increased risk with taking duloxetine for prolonged treatment in Chinese patients with OA pain compared to the acute treatment phase or other chronic pain conditions in previous duloxetine studies.

Seven PLA_DLX patients and 3 DLX_DLX patients reported at least 1 fall at the end of the extension phase. Falls have been reported with therapeutic doses of duloxetine [39]. The risk of falling appears to increase with age and appears to be related to orthostatic hypotension, concomitant medications that may induce orthostatic hypotension, medical comorbidities (such as preexisting cardiorespiratory conditions), and gait disturbances [40, 41].

One of the limitations of this study is that the extension phase was open-label and uncontrolled. Because the lack of blinding may have introduced a bias in the evaluation of maintenance of effect and of the safety profile, findings from the extension phase should be interpreted with caution. However, placebo-controlled studies in pain are always of limited duration due to ethical and practical concerns. Another limitation is that this study included only Chinese patients and excluded patients with certain psychiatric or medical disorders, so results should be extrapolated with care to the general population. Finally, the extension phase only lasted for 13 weeks. Future studies with longer duration may investigate whether the analgesic effect of duloxetine can be maintained in patients with OA pain for longer time.

## Conclusions

Chinese patients with OA pain maintained the improvement in pain measures that occurred in the 13-week placebo-controlled phase during 13 weeks of continued treatment with duloxetine. Moreover, additional pain relief was observed during the extension phase, further highlighting the importance of treatment continuation. Duloxetine was well tolerated during long-term treatment and demonstrated a safety profile similar to that observed in previous clinical trials.

## Abbreviations

5-HT: Serotonin
AE: Adverse event
ALT: Alanine aminotransferase
BPI: Brief Pain Inventory
CI: Confidence interval
CLBP: Chronic low back pain
DLX: Duloxetine
DPNP: Diabetic peripheral neuropathic pain
MMRM: Mixed model repeated measures
NE: Norepinephrine
NSAIDs: Nonsteroidal anti-inflammatory drugs
OA: Osteoarthritis
PLA: placebo
QD: Once daily
SAEs: Serious adverse events
TEAEs: Treatment-emergent adverse events
ULN: Upper limit of normal
